# An Assessment of the Performance of Different Chatbots on Shoulder and Elbow Questions

**DOI:** 10.3390/jcm14072289

**Published:** 2025-03-27

**Authors:** Mohamad Y. Fares, Tarishi Parmar, Peter Boufadel, Mohammad Daher, Jonathan Berg, Austin Witt, Brian W. Hill, John G. Horneff, Adam Z. Khan, Joseph A. Abboud

**Affiliations:** 1 Division of Shoulder and Elbow Surgery, Rothman Orthopaedic Institute, Philadelphia, PA 19107, USA; paboufadel@gmail.com (P.B.); abboudj@gmail.com (J.A.A.); 2 Penn State College of Medicine, The Pennsylvania State University, Hershey, PA 17033, USA; tarishiparmar20051@gmail.com; 3 Department of Orthopedic Surgery, The Warren Alpert Medical School, Brown University, Providence, RI 02912, USA; mohdaher1@hotmail.com; 4 Sidney Kimmel Medical College, Thomas Jefferson University, Philadelphia, PA 19107, USA; jonathan.berg@students.jefferson.edu; 5 Baylor University Medical Center, Dallas, TX 75246, USA; austin.nwitt@gmail.com; 6 Palm Beach Orthopaedic Institute, West Palm Beach, FL 33401, USA; bwhill2@gmail.com; 7 Division of Shoulder and Elbow Surgery, Department of Orthopaedics, University of Pennsylvania, Philadelphia, PA 19104, USA; jghorneff3@gmail.com; 8 Southern Permanente Medical Group, Pasadena, CA 91188, USA; adamzkhan12@gmail.com

**Keywords:** ChatGPT, AAOS ResStudy, performance, questions

## Abstract

**Background/Objectives:** The utility of artificial intelligence (AI) in medical education has recently garnered significant interest, with several studies exploring its applications across various educational domains; however, its role in orthopedic education, particularly in shoulder and elbow surgery, remains scarcely studied. This study aims to evaluate the performance of multiple AI models in answering shoulder- and elbow-related questions from the AAOS ResStudy question bank. **Methods**: A total of 50 shoulder- and elbow-related questions from the AAOS ResStudy question bank were selected for the study. Questions were categorized according to anatomical location, topic, concept, and difficulty. Each question, along with the possible multiple-choice answers, was provided to each chatbot. The performance of each chatbot was recorded and analyzed to identify significant differences between the chatbots’ performances across various categories. **Results**: The overall average performance of all chatbots was 60.4%. There were significant differences in the performances of different chatbots (*p* = 0.034): GPT-4o performed best, answering 74% of the questions correctly. AAOS members outperformed all chatbots, with an average accuracy of 79.4%. There were no significant differences in performance between shoulder and elbow questions (*p* = 0.931). Topic-wise, chatbots did worse on questions relating to “Adhesive Capsulitis” than those relating to “Instability” (*p* = 0.013), “Nerve Injuries” (*p* = 0.002), and “Arthroplasty” (*p* = 0.028). Concept-wise, the best performance was seen in “Diagnosis” (71.4%), but there were no significant differences in scores between different chatbots. Difficulty analysis revealed that chatbots performed significantly better on easy questions (68.5%) compared to moderate (45.4%; *p* = 0.04) and hard questions (40.0%; *p* = 0.012). **Conclusions**: AI chatbots show promise as supplementary tools in medical education and clinical decision-making, but their limitations necessitate cautious and complementary use alongside expert human judgment.

## 1. Introduction

ChatGPT is an artificial intelligence (AI) large language model (LLM) developed by OpenAI that was initially released in November 2022 [1]. It functions as a chatbot to simulate natural conversations by synthesizing user input and generating responses to specific words, phrases, questions, or other prompts [2]. By January 2023, ChatGPT became the fastest growing consumer application to reach 100 million users, and its widespread implementation continues to expand [3]. Since then, multiple iterations of chatbot AI programs have been developed including Google’s Gemini software (formerly known as Bard) and Microsoft’s CoPilot AI, which were released in March and November 2023, respectively [4,5].

The use of AI within the scientific community has garnered much attention recently, with numerous studies exploring the risks and benefits associated with its educational and clinical use [6,7,8,9,10,11]. A direct way to assess these AI models is by administering medical licensing exams or certified question banks. Kung et al. evaluated the performance of ChatGPT on the United States Medical Licensing Examination (USMLE) Step 1, Step 2CK, and Step 3, with reported accuracy rates > 50%, which led to near passing or passing scores on the exams [12]. A similar study by Skalidis et al. administered the European Exam in Core Cardiology to ChatGPT, which scored an average total accuracy of 58.8% across all question sources [13]. Hoffman et al. showed the newer model of ChatGPT, GPT-4, correctly answered 63.4% of the questions when given Orthopedic In-Training Examination questions (OITE) [14]. In April 2024, Vaishya et al. compared the performance of multiple chatbots including ChatGPT-3.5, ChatGPT-4, and Gemini when provided an orthopaedicorthopedic qualifying examination [15]. The results showed that ChatGPT-4 answered 65/120 (54.2%) questions correctly while ChatGPT-3.5 only answered 54/120 (45%) correctly, demonstrating a significant improvement between generations [15]. Impressively, Gemini answered all 120 questions correctly [15]. These data suggest a continuously improving performance on the part of AI software compared to previous models, which may eventually guide clinical applications.

This study aims to evaluate the performance of multiple AI models (ChatGPT-3.5, ChatGPT-4, ChatGPT-4o, Gemini, and CoPilot) in answering shoulder- and elbow-related questions from the American Academy of Orthopaedic Surgeons (AAOS) question bank, ResStudy. By doing so, we aim to provide insight into the knowledge of modern chatbots regarding different shoulder and elbow pathologies and evaluate their role as a potential learning tool for shoulder and elbow medical professionals.

## 2. Materials and Methods

The ResStudy question bank from the AAOS website was accessed in order to obtain shoulder- and elbow-related questions [16]. Questions with no figures or pictures were included in our study. The reason behind this is because the majority of chatbots do not allow for the incorporation of pictures or figures into their prompts. A total of 50 questions were chosen and included in our study. The questions were categorized according to whether they pertained to shoulders or elbows, according to explored topics (instability, adhesive capsulitis, arthroplasty/arthritis, trauma, rotator cuff, and nerve injuries) and according to explored concepts (diagnosis, management, prognosis, and anatomy). Correct answers were recorded, as well as the percentage of AAOS members who answered the questions correctly. We further used this percentage in order to further categorize the questions according to difficulty: easy (more than 75% of AAOS members answered correctly), moderate (between 50 and 75% of AAOS members answered correctly), and difficult (less than 50% of AAOS members answered correctly).

Each question, along with the possible multiple-choice answers, were provided to each of the following chatbots: ChatGPT-3.5, ChatGPT-4, ChatGPT-4o, Gemini, and CoPilot. The response of every chatbot was recorded, and the rates of correct answers were calculated. Descriptive statistics were used to report on the performance of every chatbot for the different categories (anatomic location, topic, and difficulty). A Chi-squared analysis was conducted to test for significant differences between the performance of the different chatbots in answering the questions included in our study. One-way analysis of variance (ANOVA) testing was used to compare the individual performance of the chatbots on different questions according to the level of difficulty, topics, and concepts tested. An independent t-test was used to compare the scores of the different chatbots on answering shoulder vs. elbow questions. A *p*-value of less than 0.05 was considered significant. The statistical package for the social sciences by IBM (SPSS, 2017) was used to conduct all statistical analyses in this study.

Our study is a cross-sectional study that does not involve patients, and as such, an institutional review board was not required.

## 3. Results

### 3.1. Characteristics of Included Questions

A total of 22 questions pertaining to the elbow and 28 questions pertaining to the shoulders were included in our study (*n* = 50). Instability was the most explored topic overall with 16 questions (32.0%), followed by arthritis/arthroplasty at 14 (28.0%). Management was the most commonly explored concept with 17 questions (34.0%), followed by anatomy at 16 questions (32.0%). For the shoulders, the most commonly explored topic was arthroplasty (*n* = 10, 35.7%) and the most commonly explored concepts were management and anatomy (*n* = 8, 28.6% each). For the elbow, the most commonly explored topic was instability (*n* = 10, 45.4%), and the most commonly explored concept was management (*n* = 9, 41.0%). The majority of the questions were determined to be of easy difficulty (*n* = 33, 66.0%), 13 questions (26.0%) were determined to be of moderate difficulty, and 4 questions (8.0%) were determined to be difficult questions. Table 1 shows the distribution of the included questions in our study according to relevant anatomic location, topic, concept, and difficulty.

### 3.2. Overall Performance

The average performance of all chatbots was 60.4%, scoring around 30 out of 50 questions correctly. GPT4o performed the best out of all the chatbots, providing the correct answer for 37 out of 50 questions (74.0%). This was followed by GPT 4 with 35 correct answers (70.0%), and ChatGPT 3.5 with 29 correct answers (58.0%). Gemini and CoPilot had the lowest scores with 25 correct answers each (50.0%) (Table 2). A statistically significant difference was observed when conducting Chi-Squared analysis comparing the performance of the different chatbots (*p* = 0.034). That being said, AAOS members performed better than all other chatbots; on average, 79.4% of AAOS members scored the correct answer for each question.

### 3.3. Performance According to Shoulder Versus Elbow Questions

When exploring the shoulder questions separately, ChatGPT 4o performed the best, correctly answering 22 out of 28 questions (78.5%), followed by GPT 4, which answered 20 questions correctly (71.4%). CoPilot and ChatGPT 3.5 performed the worst with only 14 correct answers each (50.0%), while Gemini answered 15 questions correctly (53.5%). On the other hand, 80.6% of the AAOS members chose the correct answer for the questions pertaining to shoulders (Table 3). When exploring elbow questions separately, ChatGPT 4o, GPT 4, and ChatGPT 3.5 all performed equally best with 15 out of 22 correct answers (68.2%). CoPilot had 11 correct answers (50.0%), and Gemini performed the worst with 10 out of 22 correct answers (45.4%). When assessing performance of AAOS members, it was observed that 77.9% answered the elbow questions correctly (Table 3). There were no significant differences in the performance of the different chatbots on shoulder questions compared to elbow questions (*p* = 0.931; MD: 0.702).

### 3.4. Performance According to Topic

The mean percentage of correct answers for the different chatbots was 71.3% for “Instability”, 80.0% for “Nerve Injuries”, 67.1% for “Arthroplasty”, 48.0% for “Rotator Cuff Tears”, 46.0% for “Trauma”, and 26.6% for “Adhesive Capsulitis” (Table 4).

For “Instability”, ChatGPT 4o performed the best with 14 out of 16 correct answers (87.5%), whereas Gemini and CoPilot performed equally worst with 9 out of 16 correct answers (56.3%). For “Nerve Injuries”, ChatGPT 3.5, GPT 4, and ChatGPT 4o answered 2 out of 2 questions correctly (100%), whereas Gemini and CoPilot answered only 1 question correctly (50.0%). For “Arthroplasty”, GPT 4 performed the best with 12 out of 14 correct answers (85.7%), whereas CoPilot performed the worst with 6 out of 14 correct answers (42.9%). For “Rotator Cuff Tears”, GPT 4 performed the best with 4 out of 5 correct answers (80.0%), whereas Gemini performed the worst with 1 out of 5 correct answers (20.0%). For “Trauma”, CoPilot and ChatGPT 4o performed equally best with 6 out of 10 correct answers (60.0%), whereas GPT 4 performed the worst with 3 correct answers (30.0%). Finally, for adhesive capsulitis, all chatbots had one out of three questions answered correctly (33.3%), except for Gemini, which had no correct answers (0%) (Table 4). When comparing the performance of the different chatbots according to topic, it was seen that the chatbots performed significantly worse on “Adhesive Capsulitis” when compared with “Instability” (*p* = 0.013), “Nerve Injuries” (*p* = 0.002), and “Arthroplasty” (*p* = 0.028).

### 3.5. Performance According to Concept

The mean percentage of correctness for the different chatbots was 71.4% for “Diagnosis”, 57.7% for “Management”, 56.3% for “Anatomy”, and 64.0% for “Prognosis” (Table 4).

For “Diagnosis” questions, GPT 4 performed the best, answering seven out of seven questions correctly (100%), whereas Copilot performed the worst with only two questions (28.5%) answered correctly. For “Management”, GPT 4o performed the best, answering 12 out of 17 questions (70.6%) correctly, whereas Gemini performed the worst with 8 (47.1%) answered correctly. Similarly, for “Anatomy”, GPT 4o performed the best with 13 out of 16 questions (81.3%) answered correctly, whereas Gemini performed the worst with only 6 questions (37.5%) answered correctly. Finally, for “Prognosis”, GPT 4 performed the best with 9 out of 10 questions (90.0%) answered correctly, whereas CoPilot performed the worst with only 5 questions (50.0%) answered correctly (Table 4). There were no significant differences between the performance of the different chatbots on the different tested concepts in our study (*p* = 0.535).

### 3.6. Performance According to Difficulty

The mean percentage correctness for the different chatbots was 68.5% for easy questions, 45.4% for moderate questions, and 40.0% for hard questions (Table 5).

For easy questions, GPT 4o performed the best, with 29 out of 33 questions (87.9%) answered correctly, whereas CoPilot performed the worst with 17 questions (51.5%) answered correctly. For moderate questions, ChatGPT 3.5 performed the best with 7 out of 13 questions (53.9%) answered correctly, whereas Gemini performed the worst with only 4 questions (30.8%) answered correctly. Finally, for hard questions, Gemini, CoPilot, and GPT 4 answered two out of four questions (50.0%) correctly, whereas ChatGPT 3.5 and GPT 4 answered one question (25.0%) correctly (Table 5). As expected, the performances of the different chatbots on easy questions was significantly better than their performances on moderate questions (*p* = 0.04) and hard questions (*p* = 0.012). There were no significant differences, however, between the performance of the chatbots on moderate questions and hard questions (*p* = 0.794).

## 4. Discussion

Our study showed that chatbots can answer around 60.4% of shoulder and elbow questions correctly, with the highest chatbot performance remaining lower than that of the general AAOS member population. Overall, GPT 4o performed the best out of the different chatbots, whereas Gemini and CoPilot had the lowest scores. There were no differences when comparing chatbot performance between shoulder and elbow questions. Chatbots performed generally well on topics like “Instability” and “Nerve Injuries”, but poorly on topics like “Adhesive capsulitis”. Moreover, questions conceptualized around diagnosis showed a relatively good performance by the chatbots overall. Finally, the chatbots performed better on easy questions than on questions with moderate or hard difficulty.

Considering the popularity and hype garnered by the creation of chatbots all over the world, the overall performance of chatbots on shoulder and elbow questions, in our study, was underwhelming. While Gemini and CoPilot scored the lowest at 50% correct questions, GPT 4o performed the best at 74% correct questions. Yet, 79.4% of the general population of AAOS members provided, on average, the correct answer, thereby outperforming the most advanced chatbot in our study, GPT 4o. Several studies in the literature have explored the performance of different chatbots on orthopaedic-related examinations and questions [16,17,18,19,20]. Similarly to our study, human examiners have performed better than chatbots on self-assessment examination questions from the American Society for Surgery of the Hand [21]. Another study explored the performance of GPT 4 on the Orthopaedic In-Training Examination and found that the chatbot correlated to the level of a third year orthopaedic resident [19]. Moreover, one study compared the performance of ChatGPT 3.5, GPT 4, and Bard Google (now known as Gemini) on orthopaedic postgraduate exam questions [15]. Interestingly, the study showed that Bard (Gemini) had superior results than when compared to ChatGPT 3.5 and GPT 4, even though our study showed the opposite [15]. This highlights the variability of chatbot performances according to question topic, delivery, and scoring.

When assessing chatbot performance according to different topics and concepts, it was seen that there were no differences in performance between shoulder and elbow questions. However, there was a particular limitation when answering questions related to “Adhesive Capsulitis”. In addition, while there were no significant differences in performance between the different concepts explored in our study, it was seen that the chatbots did poorly on questions related to “Management” and “Anatomy”. Chatbots are designed to employ natural language processing techniques in order to scrape large online databases of currently available literature in order to generate appropriate responses to different queries [22]. Hence, the performance of the different chatbots in our study is a reflection of the quality of the currently available literature, the accuracy and efficiency of the processing models utilized by the chatbots, and the clarity of the questions chatbots are asked [22]. By categorizing the different questions according to topics and concepts, and analyzing chatbot performances according to these different topics and concepts, we are able to deduce areas of particular weaknesses. According to our study, it is possible that information targeting adhesive capsulitis, as well as information dealing with shoulder and elbow “anatomy” and “management” is not of a high quality and reliability, thereby explaining the poor performance exhibited by the chatbots in our study. Perhaps focusing on publishing well-written peer-reviewed publications on these topics can help improve the knowledge of the chatbots and enhance their ability to respond to related questions.

When assessing performances according to perceived difficulty of the questions, it was seen that, on average, the chatbots performed better on easy questions than on questions with a moderate or hard difficulty. This is interesting, as it shows that the performance trends and patterns of the chatbots emulate that of the human examinees. What is considered difficult for AAOS members was also considered difficult for the different chatbots. This is warranted, as the chatbots utilize human-generated literature in order to come up with responses [22]. However, this carries the implication that the methods by which the chatbots process the literature and the way by which they perceive questions is similar to that of humans. Continuous training and updates can help improve the efficiency and knowledge of the different chatbots, leading to improved and enhanced performance. In this setting, it was shown that GPT 4o performed better than GPT 4, which in turn, performed better than ChatGPT 3.5. This reflects the aforementioned efforts in continuously improving and enhancing chatbot accuracy, reliability, and performance.

To our knowledge, this is the first study to assess the performance of different chatbots in answering shoulder- and elbow-related questions. That being said, several limitations exist. One of which is the exclusion of questions involving figures or images, which are critical components in medical diagnostics and education but were not compatible with the text-based input capabilities of the chatbots. Further, the study has evaluated the performance of these chatbots as per the current day. However, these AI models are continually evolving, with developers frequently releasing new versions, which may lead to different results over time.

## 5. Conclusions

While chatbots like ChatGPT-3.5, ChatGPT-4, GPT-4o, Gemini, and CoPilot demonstrated considerable potential in answering shoulder- and elbow-related questions from the AAOS ResStudy question bank, their performance still lagged behind that of AAOS members. Specifically, GPT-4o outperformed the others with a correct answer rate of 74%, yet it still fell short of the 79.4% average accuracy achieved by AAOS members. Chatbots excelled in areas such as “Instability” and “Nerve Injuries”, but struggled significantly with “Adhesive Capsulitis”. Furthermore, the difficulty level of the questions impacted chatbot performances, mirroring human trends with better results on easier questions. These findings underscore the continuous improvement in AI capabilities and highlight the potential of chatbots as supplementary tools in medical education and diagnostics. However, they also indicate that medical students and professionals should exercise caution when relying on these bots for accurate answers to shoulder and elbow-related questions. The results, also, emphasize the need for ongoing refinement and targeted enhancement, especially in areas where chatbot performance is currently deficient.

## Figures and Tables

**Table 1:** Distribution of Questions (*n* = 50) according to relevant anatomic location, topic, concept, and difficulty.

**Anatomic Location**		
	***n***	**%**
**Shoulder**	28	56
**Elbow**	22	44
**Topic**		
	***n***	**%**
**Instability**	16	32
**Neuro**	2	4
**Arthroplasty**	14	28
**Rotator Cuff Tear**	5	10
**Trauma**	10	20
**Adhesive Capsulitis**	3	6
**Concept**		
	***n***	**%**
**Diagnosis**	7	14
**Management**	17	34
**Anatomy**	16	32
**Prognosis**	10	20
**Difficulty**		
	***n***	**%**
**Easy**	33	66
**Moderate**	13	26
**Hard**	4	8

**Table 2:** Performance of different chatbots on shoulder and elbow questions.

	Number of Correct Questions (*n* = 50)	% Questions Correct
**GPT 4o**	37.0	76.0
**GPT 4**	35.0	70.0
**ChatGPT 3.5**	29.0	58.0
**Gemini**	25.0	50.0
**CoPilot**	25.0	50.0

**Table 3:** Comparison of chatbot performance between shoulder-related and elbow related questions.

		ChatGPT 3.5	Gemini	CoPilot	GPT 4	GPT 4o	AAOS Members
Elbow	Number of Correct Answers	15	10	11	15	15	N/A
	Percentage of Correct Answers	68.18	45.45	50.00	68.18	68.18	80.60
Shoulder	Number of Correct Answers	14	15	14	20	22	N/A
	Percentage of Correct Answers	50	53.5	50	71.4	78.5	77.9

**Table 4:** Performance of different chatbots on shoulder and elbow questions according to topic and concept.

		Total Questions	ChatGPT 3.5 % Correct	Gemini % Correct	CoPilot % Correct	GPT 4 % Correct	GPT 4o % Correct	Overall Mean % Correct
**Topic**	**Instability**	16	75	56.25	56.25	81.25	87.5	71.25
	**Neuro**	2	100	50	50	100	100	80
	**Arthroplasty**	14	57	71.4	42.9	85.7	78.6	67
	**Rotator Cuff Tear**	5	40	20	40	80	60	48
	**Trauma**	10	40	40	60	30	60	46
	**Adhesive Capsulitis**	3	33.3	0	33.3	33.3	33.3	26.7
**Concept**	**Diagnosis**	7	71.43	71.43	28.57	100	85.71	71.43
	**Management**	17	58.82	47.06	52.94	58.82	70.59	57.65
	**Anatomy**	16	50	37.5	56.25	56.25	81.25	56.25
	**Prognosis**	10	60	60	50	90	60	64

**Table 5:** Performance of different chatbots on shoulder and elbow questions according to difficulty.

Difficulty	Total Questions	ChatGPT 3.5 % Correct	Gemini % Correct	CoPilot % Correct	GPT 4 % Correct	GPT 4o % Correct	Overall Mean % Correct
**Easy**	33	63.64%	57.58%	51.52%	81.82%	87.88%	68.48%
**Moderate**	13	53.85%	30.77%	46.15%	46.15%	50.00%	45.38%
**Hard**	4	25%	50.00%	50.00%	50.00%	25.00%	40.00%

## Data Availability

The data presented in this study are available on request from the corresponding author (data are not publicly available due to privacy or ethical restrictions).
